# Non-melanoma Skin Cancers in Patients on Hydroxyurea for Philadelphia Chromosome-Negative Myeloproliferative Neoplasms: A Systematic Review

**DOI:** 10.7759/cureus.16978

**Published:** 2021-08-07

**Authors:** Divya R Gavini, Dhairya J Salvi, Prutha H Shah, Davuluri Uma, Jun Hee Lee, Pousette Hamid

**Affiliations:** 1 Internal Medicine, California Institute of Behavioral Neurosciences & Psychology, Fairfield, USA; 2 Neurology, California Institute of Behavioral Neurosciences & Psychology, Fairfield, USA

**Keywords:** hydroxyurea, skin cancers, squamous cell carcinoma, basal cell carcinoma, philadelphia chromosome negative, myeloproliferative neoplasms

## Abstract

Hydroxyurea (HU) or hydroxycarbamide is a cytotoxic antimetabolite widely used to treat Philadelphia chromosome-negative Myeloproliferative Neoplasms (Ph-MPN) like Polycythemia Vera (PV), Essential Thrombocythemia (ET), and Primary Myelofibrosis (PMF). Patients with Ph-MPN are at an increased risk of Non-melanoma skin cancers (NMSC). The cause of this finding remains uncertain. In this systematic review, we would like to know if chronic use of HU in this population is responsible for the sudden onset of NMSC. The results obtained will help the patients and clinicians with early diagnosis of cutaneous lesions and in optimizing the current treatment options for MPN. We conducted a multi-database literature search, applied eligibility criteria and quality assessment tools to the studies extracted, with an intention to include only fair to high-quality articles. We analyzed six observational studies and four traditional reviews. Two out of 10 studies concluded that no relationship exists between the incidence of NMSC and HU. The remaining eight studies indicated the association. According to these studies, the possible risk factors include old age, excessive exposure to sunlight, higher doses, and prolonged HU therapy duration. Ultraviolet (UV) radiation and HU play a combined role in carcinogenesis. Periodic dermatologic screening is essential in these patients. Prompt biopsy and accurate diagnosis can prevent the progression of cancer and decrease the associated morbidity and mortality. True incidence and causation cannot be ascertained due to the scarcity of research on this topic. Multi-center prospective studies in large groups of Ph-MPN patients are recommended to determine the temporal relationship between NMSC and HU treatment.

## Introduction and background

Hydroxyurea (HU), also called hydroxycarbamide, is a non-alkylating antimetabolite identified in 1869 by Dresler and Stein [1]. It is used to treat sickle cell anemia, myeloproliferative disorders, hypereosinophilic syndrome, and refractory psoriasis [2-6]. Given the safety and efficacy of tyrosine kinase inhibitors, HU is the drug of choice only in Philadelphia chromosome-negative (Ph-) Myeloproliferative Neoplasms (MPN). HU inhibits the enzyme ribonucleotide reductase and prevents the formation of deoxyribonucleotides from ribonucleotides in the S phase of the cell cycle [2-3]. It thus inhibits cellular proliferation and finds its use as a cytoreductive agent in Ph-MPN [3].

HU is considered to be well-tolerated, but a quick literature search highlights the varied presentation of cutaneous side effects of this drug [4]. The adverse cutaneous reactions range from alopecia, hyperpigmentation, melanonychia, xerosis, keratoderma, ulcers, dermatomyositis-like eruptions, actinic keratosis to various types of malignancies [7-8]. Chronic nonhealing ulcers, dermatomyositis-like eruptions, and actinic keratosis are pre-malignant, and if not recognized, can progress to full-blown malignancy. The cutaneous malignancies include Squamous cell carcinoma (SCC), Basal cell carcinoma (BCC), and Merkel cell carcinoma (MCC) [7,9]. According to the reported studies, these carcinomas were observed in patients on medication with this drug for a longer duration [7-8]. Patients with Ph-MPN are usually on a relatively high steady dose for an extended period of time.

Ph-MPN mainly includes three disorders: Polycythemia Vera (PV), Essential Thrombocythemia (ET), and Primary Myelofibrosis (PMF). Recent studies state that patients with Ph-MPN are at increased risk of secondary cancers [10-11]. These secondary cancers could be either hematological (leukemia, lymphoma) or non-hematological (skin, lung, kidney, and thyroid cancers) in nature. The risk of developing non- hematological solid cancers is 1.5 to 3 times more in patients with MPN than in the general population [10]. The risk of Non-Melanoma skin cancers (NMSC) is particularly increased among non-hematological secondary cancers [10-11]. Multidisciplinary screening is required in these patients to decrease the specified risk. According to some researchers, arterial thrombosis, which is very common in these patients, could be a possible risk factor [12]. It is not evident if the pathophysiology associated with MPN plays a role in skin cancers or is caused by antineoplastic drugs used in the treatment. HU is the most common and widely used drug for Ph-MPN. In this review, we would like to find out if there is a clear association between NMSC and HU therapy. This study is in the best interest of hematologists, dermatologists, and patients with Ph-MPN. The results obtained can help with early diagnosis and optimize the current treatment options, thereby decreasing the disease burden and increasing patients' quality of life.

According to a report published in 2014, the health care resource utilization associated costs for patients with MF, PV, and ET in the United States is 5.3, 1.8, and 3.6 times higher, respectively, as compared to age and gender-matched people without Ph-MPN [13]. Increased disease reporting through cancer registries and large population studies can better comprehend the disease process and prevent complications. In this systematic review, we plan to methodically group and meticulously analyze all available evidence. Our objectives are: to understand the interrelation between HU and NMSC in patients with Ph-MPN; to explain the pathogenetic mechanisms; to look for any other contributing risk factors; and to determine if dermatological surveillance is required in patients with MPN on hydroxyurea. 

## Review

Methodology

This Systematic Review is according to the Preferred Reporting Items for Systematic Reviews and Meta-Analyses (PRISMA) guidelines [14].

Search Strategy

We conducted a comprehensive multi-database search to study the association of HU in causing skin cancers in patients with MPN. The data were collected from PubMed, PubMed Central (PMC), Google Scholar, and Science Direct databases. The first strategy was to search using regular keywords. The second search strategy was to include both regular and MeSH keywords. Related keywords were combined using OR, and unrelated keywords were joined using AND (Boolean Search). We followed a building block approach. The search was conducted on April 12, 2021. The first search strategy yielded 19,000 articles in Google Scholar, 4,444 articles in Science Direct, and 429 articles in PMC. We extracted 113 papers from PubMed using second search strategy, which is as follows: Myeloproliferative Disorders OR Polycythemia Vera OR Essential Thrombocythemia OR Myelofibrosis OR ("Myeloproliferative Disorders/complications"[Mesh] OR "Myeloproliferative Disorders/drug effects"[Mesh] OR "Myeloproliferative Disorders/drug therapy"[Mesh]) AND Hydroxyurea OR Antimetabolite OR Siklos OR hydroxycarbamide OR Hydrea OR Droxia OR ("Hydroxyurea/administration and dosage"[Mesh] OR "Hydroxyurea/adverse effects"[Mesh] OR "Hydroxyurea/ physiology"[Mesh] OR "Hydroxyurea/toxicity"[Mesh]) AND Skin Cancer OR Cutaneous Malignancy OR Squamous cell carcinoma OR Basal cell carcinoma OR ("Skin Neoplasms/chemically induced"[Mesh] OR "Skin Neoplasms/diagnosis"[Mesh] OR "Skin Neoplasms/epidemiology"[Mesh]).

Eligibility Criteria

The inclusion criteria for our analysis were: only systematic reviews, traditional reviews, meta-analyses, and observational studies (case-control and cohort studies); all relevant articles published after January 1, 2000, to April 12, 2021, in the English language; articles on human subjects; including articles with free access to their full text; non-melanoma skin cancers as the outcome of interest, and HU specified in the intervention/treatment group in the included studies. Case reports and case series were excluded from the review.

Data Extraction and Study Selection

Two authors independently screened all the collected articles based on the title and abstract of the article. Related references from all included studies were also screened. Those articles that fulfilled the eligibility criteria were subjected to quality assessment tests.

Quality Assessment Tools

Intending to include fair to high-quality studies, two reviewers independently used the New Castle-Ottawa questionnaire for observational studies and the scale for assessing narrative review articles (SANRA) for traditional reviews. We found no systematic reviews and meta-analyses after a detailed search on this topic.

Results

Literature Search

**Figure 1:**
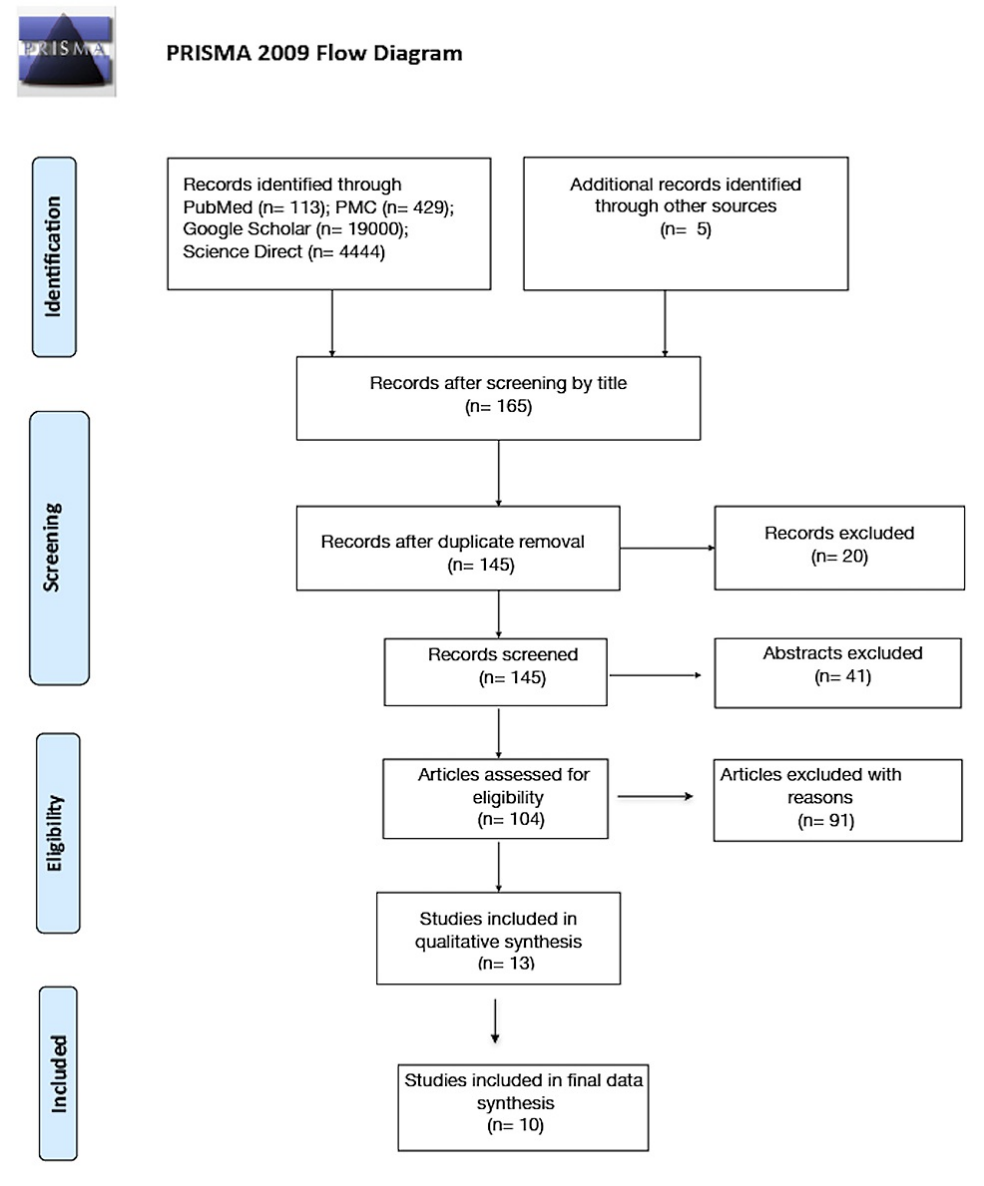
PRISMA flow diagram PRISMA: Preferred Reporting Items for Systematic Reviews and Meta-Analyses [14].

A flow diagram for identification, screening, eligibility, and inclusion of studies is shown in Figure 1. We retained a total of 145 articles after screening by the title of the study and duplicate study removal. Forty-one articles were excluded after screening by abstract. We included 13 out of 104 papers after scanning through the inclusion criteria. These 13 articles were subjected to a quality appraisal, and three of them which were of low quality were excluded. We had a final number of 10 papers that met all the required criteria.

Study Designs

We included a total of 10 articles in our study: five retrospective cohort studies, four traditional reviews, and one nested case-control study. The characteristics of each study are outlined in Table 1.

**Table 1:** Characteristics of the studies included in this systematic review AK- Actinic Keratosis; SCC- Squamous cell carcinoma; BCC- Basal cell carcinoma; NMSC- Non-melanoma skin cancers; HU- Hydroxyurea; Ph-MPN- Philadelphia chromosome-negative Myeloproliferative Neoplasms; ET- Essential Thrombocythemia; n/r- not reported.

AUTHOR, YEAR	TYPE OF STUDY	PATIENTS INCLUDED IN THE STUDY	PATIENTS ON HU	OUTCOME OF INTEREST	PATIENTS WITH OUTCOME	AGE ( in years)	DOSE AND DURATION	CONCLUSION
Antonioli et al. 2012 [15]	Retrospective Cohort	3411 with MPN	3411	AK, SCC, Basalioma	10	Median age- 65 years	AK- 1388 grams and 46 months; Basalioma - 1318 grams and 60 months of exposure	Severe toxicities are seen in a small proportion of patients with a longer duration of HU exposure
Barbui et al. 2019 [11]	Nested Case-Control Study	1881 with Ph-MPN	1316 (irrespective of the line of treatment)	SCC, BCC	127	n/r	Dose- n/r; Duration- 36 months of exposure	HU has a two-fold risk of NMSC on stratified cancer-specific analysis
Bulte et al. 2020 [7]	Traditional Review	—	—	AK, SCC, BCC, Bowen’s Disease, Keratoacanthomas, Squamous dysplasia, Merkel cell carcinoma.	20	n/r	Mean Latency Period- 79 months	NMSC is seen in a substantial proportion of patients on HU
Cantisani et al. 2019 [16]	Traditional Review	—	—	AK, SCC, BCC	24 (from other studies); 9 (own observation)	Age Range 59-82 years (from other studies); Mean- 77+/- 6.5 years (own observation)	Cumulative Dose Range- 1- 5600 grams; Duration Range- 6 months to 15 years (from other studies)	NMSC is associated with HU treatment
Kissova et al. 2014 [17]	Retrospective Cohort	172 with Ph-MPN	66	SCC, BCC	9	Median - 67 years	Median Dose- 1394 grams Median Duration- 5 years of exposure	Patients on HU are at increased risk of skin cancers (including malignant melanoma)
Malato et al. 2020 [18]	Traditional Review	—	—	AK, SCC, BCC	68	Median- 70.6 years	Median Dose- 1.25 grams; Median Duration- 75 months of exposure	Possible risk of NMSC in patients on HU
Radaelli et al. 2008 [19]	Retrospective Cohort	331 with ET	116 (monotherapy)	NMSC	0 (11 had non- hematological cancers but no skin cancers)	Median- 61 years	Dose- n/r; Median Duration- 108 months of follow up	HU has no association with the occurrence of secondary solid cancers
Sanchez-Palacios et al. 2004 [20]	Traditional Review	—	—	AK, SCC, BCC, Squamous dysplasia	17 (from other studies); 2 (own observation)	Range- 50-83 years	Dose Range- 650 - 5600 grams; Duration- 2-13 years	NMSC is associated with HU treatment
Santoro et al. 2017 [21]	Retrospective Cohort	1026 with ET	641	Skin cancers other than Basalioma	5	Median- 62 years	Dose- n/r; Median Duration- 112 months of exposure; 6 years of follow up	No statistical significance between HU and occurrence of secondary malignancies
Verner et al. 2014 [22]	Retrospective Cohort	188 with Ph-MPN	149	BCC, SCC, Metastatic SCC	51	Mean- 79 years	Dose- 730 mg; Duration- 5.5 years of exposure	NMSC is common in patients with Ph-MPN on HU

Outcomes

Eight out of 10 studies reported an association between HU and NMSC [7,11,15-18,20,22]. Two studies found no statistical significance between HU and the development of secondary solid cancers (including cutaneous malignancies) [19,21]. Both of these studies were restricted to patients with ET.

Discussion

Study Analysis

The first case of HU-associated SCC was reported in 1991 [23]. Numerous case reports were published later on claiming causality. In this review, we have included observational and review articles to demonstrate if an association exists between HU therapy and NMSC.

The coalescent dry erythematous patches that were seen after treatment with HU were initially termed photodermatitis, or dermatomyositis-like reaction, or lichen planus like eruption. They were assumed to take a benign course, but most of them progressed into an indolent SCC. Sanchez-Palacios et al. introduced the term "HU-associated squamous dysplasia" for the first time to describe these pre-malignant lesions revealing the seriousness of the condition [20]. Patients with Ph-MPN on HU developing skin cancers are usually elderly individuals [20,22]. The minimum median age of patients included in our studies is at least 61 years. There was no clear-cut association reported between the incidence and gender. Both males and females are equally susceptible, although one study reported a male predominance [22]. The majority of the individuals in the studies were on high doses of HU for a long duration. The range for dose and duration of exposure in our case population was 730 mg-5600 gm of HU for a period of six months to fifteen years. This is consistent with the extremely low incidence of skin cancers in sickle cell patients on relatively low doses of HU for a shorter duration [24]. Most lesions improved or remained stable after the drug withdrawal [7,20]. In very few cases, tumor recurrence was seen several years after discontinuation of HU [16]. Antonioli et al. described worsening of lesions and progression to SCC on continuing the HU treatment in three patients [15]. These cancers are predominantly seen on the scalp, ears, neck, hands, and feet [15-18,22]. This shows that excessive exposure to sunlight may play a contributory role in carcinogenesis.

All review articles implied a definite association between NMSC and HU treatment after an in-depth critical evaluation of every reported case [7,16,18,20]. A total of 140 cases reported from all the reviews were included in our study. Two out of six observational studies inferred that HU does not cause NMSC [19,21]. The median dose of HU was not reported in both of these studies. Santoro et al. observed similar risk in untreated patients and concluded that the deranged immune system and the ongoing inflammation in MPN patients might be the cause for the occurrence of secondary cancers, rather than being the effect of any particular drug [21]. Barbui et al. observed a two-fold risk of NMSC in patients on HU on stratified cancer-specific analysis (OR- 2.28, 95% CI- 1.15-4.15) [11]. The percentage of patients with NMSC in the remaining four observational studies, which stated an association, is listed below in Table 2.

**Table 2:** Results obtained from observational studies HU- Hydroxyurea; NMSC- Non-melanoma skin cancer

Study	Patients on HU	Patients with NMSC	Percentage of patients on HU with NMSC
Antonioli et al. 2012 [15]	3411	10	0.29%
Barbui et al. 2019 [11]	1316	127	9.6%
Kissova et al. 2014 [17]	66	9	13.6%
Verner et al. 2014 [22]	149	51	34.2%

A negligible percentage (0.29%) of patients on HU developed NMSC in the study led by Antonioli et al. [15]. Two studies reported a significant number of cases as 9.6% and 13.6% of patients on HU, respectively [11,17]. According to Kissova et al., the most common secondary non-hematological cancers reported in the study group were skin cancers, and all of them were of the HU group [17]. However, the median age and the median follow-up period were longer in patients on HU. Verner et al. identified an immense risk, but it should be noted that there is the highest incidence of NMSC in Australia [22,25].

Study Interpretation

It is evident from the above studies that there might be a possible risk of NMSC in elderly patients with Ph-MPN on high doses of HU exposed for a very long duration, especially in the sun-exposed areas. Excessive exposure to UV radiation and older age by themselves are risk factors of NMSC. Thus, they are considered confounding risk factors [20]. Withdrawal of the culprit drug may not always be possible in all patients because of the high risk of sudden thrombotic events. So the treatment options must be tailored accordingly. Though skin cancers are not mentioned, there are cohort studies conducted on a large scale detailing the mucocutaneous toxicity in patients on HU [26-27]. The severe toxicities include painful recurrent skin ulcers (most commonly seen in the perimalleolar area), aphthous ulcers, and non-ulcerative lesions with erythema and skin infiltration [26]. The incidence of cutaneous adverse effects of HU was two-fold higher in prospective studies as compared to retrospective studies [27]. This outcome enlightens us to the fact that educating patients about all the possible side effects is invaluable. Dermatologic screening is required in patients who are taking HU for a prolonged duration. The findings of this systematic review signify that early detection and biopsy of suspicious lesions are crucial. Prompt diagnosis of precancerous lesions helps in decreasing the disease severity, healthcare resource utilization, and associated costs.

Pathogenesis

HU not only inhibits DNA synthesis but also inhibits DNA repair. It increases the number of breaks in the DNA and also causes the strands to remain open longer. It decreases the DNA polymerase activity, thereby slowing the polymerization rate at the repair sites [28]. Patches of p53 mutant keratinocytes are seen in the dermo-epidermal junction and hair follicles. UV irradiation increases the number and size of these p53 mutant clones. p53 is a tumor suppressor gene, and the mutant interferes with the cellular proofreading. Therefore, the mutant clones have a high survival rate and are immune to the natural process of apoptosis [29-31]. Stem cell compartments act as physical barriers preventing the expansion of clones. UV-B light exposure activates the imprisoned clones of p53 mutant keratinocytes and enables them to colonize the adjacent compartment [32]. This forms the basis of photocarcinogenesis. Thus, sunlight acts as both tumor initiator and promoter [31]. Keratinocytes are cells that have high turnover. Increased mutant clones along with impaired DNA synthesis and repair of cells in the basal layer of the epidermis are the reasons for the explosive onset of skin tumors. HU and exposure to UV radiation play a combined role in pathogenesis. Therefore, patients on chronic HU medication must be advised to avoid excess outdoor activity, use some sorts of photoprotective barriers and chemopreventive agents like oral retinoids [7,16].

Study Limitations

This systematic review has multiple limitations. We included only those articles that met our inclusion criteria, resulting in 10 high-quality retrospective descriptive studies and traditional reviews. We could not determine the actual causation and incidence due to the study designs. The controls included in the studies may be subjected to selection bias. Out of six observational studies, study populations of four studies were from Italy, one from the Czech Republic, and one from Australia [11,15,17,19,21-22]. Therefore, most cases were confined to one particular geographical location, hence the findings of these studies cannot be generalized.

Future Recommendations

The level of evidence from the retrospective studies is inferior in comparison to prospective studies. The temporal relationship between the development of NMSC and chronic use of HU can only be addressed with prospective studies. To draw distinct conclusions, studies recruiting a large group of patients irrespective of age, sex, or location, and newly diagnosed with Ph-MPN initiated on HU must be conducted. Cancer registries must be well maintained in all countries so that further research can throw light on such unknown effects.

## Conclusions

We cannot draw precise conclusions from the observational studies due to their varied results. But the majority of the studies included in this systematic review found a possible increased risk of NMSC in patients with Ph-MPN on HU. One should also consider the potential role of HU as a causative drug if any cancerous skin lesion is identified in these patients. Though not proven, the associated risk factors could be increased age, exposure to UV light, dose, and duration of HU treatment. p53 mutant keratinocyte clones found due to excessive sun exposure along with HU-induced impaired cell repair mechanisms favor the rapid onset of skin cancers. Clinicians and patients must be well aware of this alarming complication. Patients on HU for Ph-MPN must be informed to report any severe progressing cutaneous reaction. Periodic dermatological surveillance is essential in this patient group. Early diagnosis by biopsy not only prevents the tumor progression to an advanced stage but also aids with treatment. Large prospective multi-center studies are required to uncover the incidence, morbidity, and mortality in patients with Ph-MPN on HU developing NMSC.
